# Size and shape matter: The impact of voxel geometry on the identification of small nuclei

**DOI:** 10.1371/journal.pone.0215382

**Published:** 2019-04-12

**Authors:** Martijn J. Mulder, Max C. Keuken, Pierre-Louis Bazin, Anneke Alkemade, Birte U. Forstmann

**Affiliations:** 1 Integrative Model-Based Cognitive Neuroscience Research Unit, University of Amsterdam, Amsterdam, The Netherlands; 2 Experimental Psychology, Utrecht University, Utrecht, the Netherlands; 3 Department of Neurology, Max Planck Institute for Human Cognitive and Brain Sciences, Leipzig, Germany; University at Buffalo, UNITED STATES

## Abstract

How, and to what extent do *size* and *shape* of a voxel measured with magnetic resonance imaging (MRI) affect the ability to visualize small brain nuclei? Despite general consensus that voxel geometry affects volumetric properties of regions of interest, particularly those of small brain nuclei, no quantitative data on the influence of voxel size and shape on labeling accuracy is available. Using simulations, we investigated the selective influence of voxel geometry by reconstructing simulated ellipsoid structures with voxels varying in shape and size. For each reconstructed ellipsoid, we calculated differences in volume and similarity between the labeled volume and the predefined dimensions of the ellipsoid. Probability functions were derived from one or two individual raters and a simulated ground truth for reference. As expected, larger voxels (i.e., coarser resolution) and increasing anisotropy results in increased deviations of both volume and shape measures, which is of particular relevance for small brain structures. Our findings clearly illustrate the anatomical inaccuracies introduced by the application of large and/or anisotropic voxels. To ensure deviations occur within the acceptable range (Dice coefficient scores; DCS > 0.75, corresponding to < 57% volume deviation), the volume of isotropic voxels should not exceed 5% of the total volume of the region of interest. When high accuracy is required (DCS > 0.90, corresponding to a < 19% volume deviation), the volumes of isotropic voxels should not exceed 0.08%, of the total volume. Finally, when large anisotropic factors (>3) are used, and the ellipsoid is orthogonal to the slice axes, having its long axis in the imaging plane, the voxel volume should not exceed 0.005% of the total volume. This allows sufficient compensation of anisotropy effects, in order to reach accuracy in the acceptable range (DCS > 0.75, corresponding to >57% volume deviation).

## Introduction

To study the relationship between the structure and function of the brain, it is important to identify the individual anatomical structures and their borders accurately [1]. Additionally, knowledge on the variability in location and shape of small subcortical nuclei, such as the subthalamic nucleus (STN) and the internal segment of the globus pallidus (GPi) is informative for clinical procedures such as deep brain stimulation (DBS) surgery [2,3]. Visualizing and delineating these small nuclei is of great importance for invasive procedures such as DBS in which electrodes are lowered deep into the brain to alleviate disease specific symptoms of movement disorders, such as essential tremor, Parkinson’s disease and dystonia. To accurately plan the trajectory of DBS electrodes, individual anatomical detail is necessary that can be obtained using non-invasive magnetic resonance imaging (MRI; [4])

With MRI, samples are taken from an object in world space which are reconstructed into an image at a certain resolution [5]. A large proportion of the studies presented in the literature have acquired anatomical MR images at a >1.0mm isotropic resolution. These scans are, in many cases, used to determine sub-millimeter borders between brain structures. Even when using ultra-high field (7T or higher) MRI, the average voxel volume of structural scans used to identify subcortical nuclei is 1.09mm^3^ [6]. These scans are likely to show fuzzy borders between brain structures, since the MR signal of a voxel located at the interface of two structures represents a mixture of signals of those two structures. Averaging of the signal decreases the visibility of the borders. This phenomenon is known as the partial volume effect (PVE). PVEs can substantially influence the delineation of smaller brain structures.

Intuitively, acquisition of high spatial resolution will benefit the reliability of the delineation of small subcortical nuclei. Such resolution can be achieved by increasing the acquisition matrix, reducing slice thickness, and/or decreasing the Field of View (FoV). The effects of these parameter optimizations negatively affect the signal-to-noise ratio (SNR), which reduces with decreasing voxel volume [5]. The reduced SNR can be partially compensated by increasing the number of repetitions, thereby increasing scanning time, potentially introducing a practical limitation. In practice, there is a trade-off between voxel resolution and quality of an MR image.

In addition to the voxel volume, the shape of the voxel is important. Commonly, the resolution in the z-direction (i.e. slice thickness) is traded against a higher in-plane (x,y) resolution which can result in thicker slices and higher SNR due to the increasing voxel volume [5]. Such anisotropic voxel sizes are often used to identify small brain structures in plane. Previous studies from our group have shown that 60.9% of the ultra-high field MRI visualizing the subcortex use anisotropic voxels [6]. Acquisition of anisotropic voxels allows a high in-plane resolution, thereby facilitating the identification of small brain structures in a single plane, in a more time efficient manner as compared to the acquisition of isotropic voxels. Increased scanning times to obtain isotropic voxels would increase costs, and are more prone to motion artifacts, since the participant is required to lie still for an extended period of time [7]. The latter particularly affects elderly subjects and patients with movement disorders, such as Parkinson’s disease [8]. Many studies focusing on elderly or patient populations use anisotropic scans (e.g. [9,10]). The trade-off between in-plane resolution and slice thickness could potentially affect the delineation of small brain structures. A large PVE in the z-direction negatively affects edge detection while potentially affecting volume estimations (e.g. [11]).

It is thus generally accepted that in-plane voxel size and slice thickness can affect the volumetric properties of a region of interest (ROI) especially for small brain nuclei. However, to our knowledge, there is no systematic study showing the quantitative effects of voxel size and shape on labeling accuracy.

One approach would be the application of phantoms for this purpose (e.g. [12]). Systematically changing the position of a phantom for 100 positions, with 5 different voxel sizes, and 6 different levels of anisotropy would however, result in 3000 scans. Alternatively, the use of numerical phantoms facilitates quantitative comparisons between a large range of systematically changing MR parameters. This approach allows simulations of reconstructed objects, and provides an appropriate method to address our research question. An anatomically relevant numerical phantom is the 3D extension [13] of the 2D Shepp-Logan head phantom which consists of different ellipsoids, varying in their position and signal intensity [14]. 3D printing techniques have been applied to validate this 3D numerical phantom using MRI [12].

In our MRI simulation study, we used a simplified version of the 3D Shepp-Logan numerical phantom and created a series of ellipsoid structures. Each structure was created at a different in-plane resolution, with varying slice thickness. We used these ellipsoids to investigate the selective influences of the in-plane voxel size and slice thickness on volume estimations of small structures by testing the differences in volume and shape-similarity between the labeled volume and the real dimensions of the simulated ellipsoid.

For the labeling process, we used three different simulated raters: a *liberal*, *joint*, and *optimal* rater. For the *liberal* and *joint* raters, edge voxels were included based on a probability function that was derived from a single, liberal human rater and the conjunct rating of two human raters, respectively. For the *optimal* rater, the edge voxels were included to the point that the ratio between the labeled volume and the predefined dimensions of the ellipsoid approached 1.

## Materials & methods

### MRI volume simulations

Volumes were generated using a modified version of the 3D analytical MRI phantom in the Fourier domain [13]. First, an ellipsoid was generated in image space, with a FoV of 36.0 x 36.0 x 36.0mm. This FoV was chosen to accommodate the final matrix sizes of the reconstructed images. This allowed a variation of combinations to fit the volume without incorporation of any interpolation effects. Note that, although the size of the FoV is not typical for most neuroimaging studies, in this study it serves only to cover the volume of interest while still being able to reconstruct ellipsoids with varying in-plane voxel size and slice thickness.

**Fig 1:**
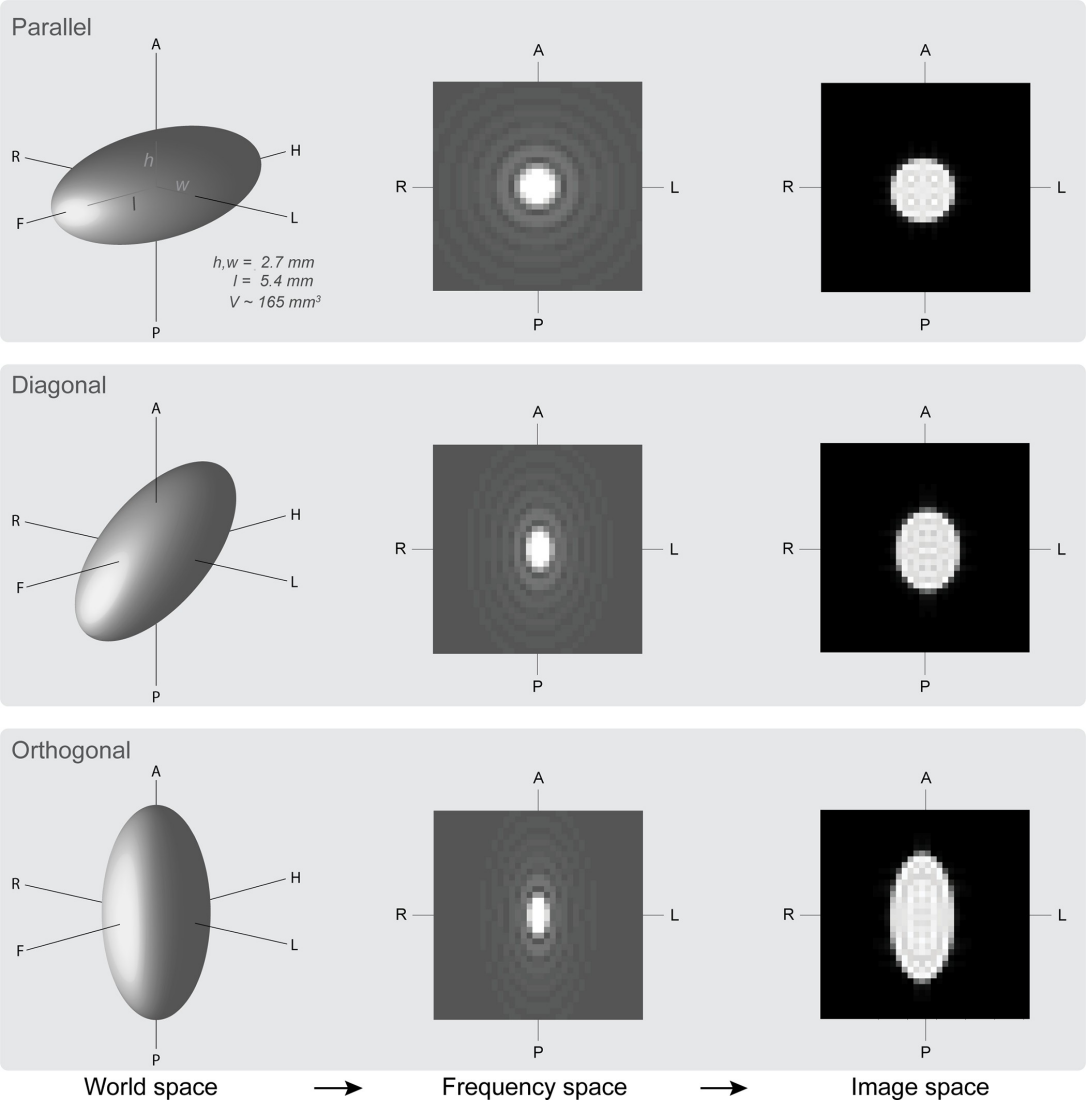
Simulation pipeline. Representation of the simulations in which ellipsoids in world space are reconstructed into MR volumes of different resolutions (image space). Orientations were chosen to resemble circumstances in the MRI scanner. The in-plane (*x*,*y*-dimension) resolution corresponds to a slice acquired along the scan-direction H-F (slice axis *z*). Anisotropy (slice thickness) was varied across the slice axis. The long axis of the ellipsoids was either placed parallel, diagonal or orthogonal to the slice axis. Figs for both frequency and image space represent the mid-slice of the reconstructed volume from sagittal view. Here reconstructed images were simulated with a voxel size of 0.5mm isotropic. A = anterior; F = feet; H = head; h = height; L = left; l = length; P = posterior; R = right; V = volume; w = width.

100 ellipsoids were created with a predefined volume of 164.9mm^3^, with radii of length (l) = 2.7mm, width (w) = 2.7mm and height (h) = 5.4mm (corresponding to a proportion of l = w = 0.075 and h = 0.15 of the FOV, respectively; see Fig 1). This volume was chosen as it is close to the reported volume estimates of the STN and the GPi, two frequently used targets for DBS in Parkinson’s Disease (see Table 1).

**Table 1:** *Post mortem* hemispheric volume estimates for a number of deep brain stimulation targets. *STN*: *subthalamic nucleus; GPi*: *globus pallidus internal segment*.

Structure	Author	Method	Volume (mm^3^)
STN	Fussenich, 1967 [15]	Microscopy	64.0
	Hardman et al., 2002 [16]	Microscopy	120.0
	Levesque & Parent, 2005 [17]	Microscopy	175.0
	Lange, Thorner, Hopf, & Schroder, 1976 [18]	Microscopy	141.0
	Massey et al., 2012 [19]	MRI 9.4T	106.0
	Nowinski, Belov, Pollak, & Benabid, 2005 [20]	Atlas reconstruction	174.0
	Plantinga et al., 2016 [21]	MRI 7.0T	100.5
	Bonin & Shariff, 1951 [22]	Microscopy	157.0
	Weiss et al., 2015 [23]	MRI 7.0T	109.0
	Yelnik & Percheron, 1979 [24]	Microscopy	180.0
	Zwirner et al., 2017 [25]	MRI 3.0T	99.0
		Microscopy	131.0
		*Average*	*129*.*7 (SD 36*.*5)*
GPi	Lange et al., 1976 [18]	Microscopy	494.0
	Mai, Majtanik, & Paxinos, 2015 [26]	Microscopy	263.5
	Plantinga et al., 2016 [21]	MRI 7.0T	271.8
	Yelnik & Percheron, 1979 [24]	Microscopy	478.0
		*Average*	*376*.*8 (SD 126*.*3)*

To simulate between-subject variations, 100 random variations of small rigid-body motion was applied to each ellipsoid (translation varied between -4.0 and +4.0mm, and rotation varied between -8.0˚ and +8.0˚). In addition, to test whether the orientation of the ellipsoid interacted with slice thickness, we created ellipsoids in which the longest axis was *parallel*, *diagonal* or *orthogonal* to the slice axis (H-F axis; see Fig 1).

Each of the 300 ellipsoids (100 objects, three orientations each) was reconstructed in an MRI volume with a predefined in-plane resolution and slice thickness. The k-space signal was analytically derived from the ellipsoid in specific resolution, and stored in the Fourier domain [13]. Next, the volume was reconstructed in image space with the specific resolution. The reconstruction resulted in an image with comparable PVE as observed in MR images, which allowed us to test the effects of voxel size and shape on volume properties of a labeled region.

**Fig 2:**
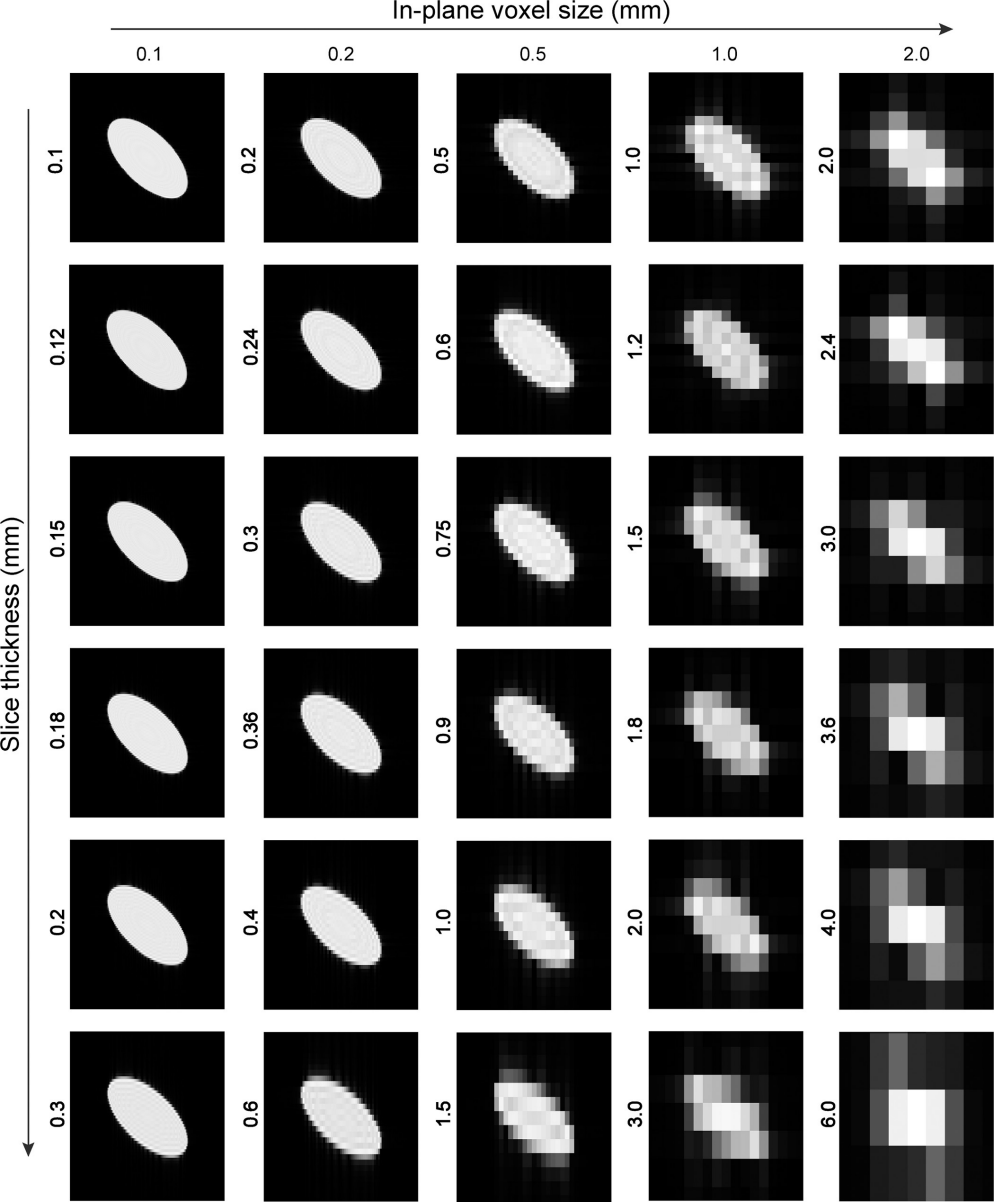
Sagittal view of central-slice examples from simulated ellipsoids. Each generated ellipsoid was reconstructed from a k-space transformation using a different in-plane voxel size (mm) and a varying slice thickness (mm). Columns represent the in-plane (xy) voxel size, ranging from left to right with a size of .1, to .2, .5, 1.0, and 2.0mm. Rows represent slice thickness that are proportional to the in-plane resolution, ranging from top to bottom with a factor of 1x (isotropic) to 1.2x, 1.5x, 1.8x, 2x and 3x the in-plane voxel size. For illustration purposes, sagittal slices were used to show the effect of voxel size and slice thickness on the diagonal orientation.

For the resolution, we used five isotropic in-plane (x, y dimension) sizes (0.1, 0.2, 0.5, 1.0 and 2.0mm) in combination with six slice thicknesses that were proportional to the in-plane size (1.0, 1.2, 1.5, 1.8, 2.0, and 3.0 times the in-plane resolution). As a result, we obtained 30 different reconstructed versions of each ellipsoid varying in its resolution. Ellipsoids had three different orientations, each with a 100 unique rigid body transformations, totaling to 9000 volumes (see Fig 2 for an illustration of the 30 different versions of an ellipsoid with an diagonal orientation).

### Raters

#### Manual labeling

Labeling a region of interest (ROI) can be done manually or automatically [27]. To our knowledge, no algorithms used for automated detection of edges of small regions outperforming manual labeling have been published [28,29]. However, manual labeling is extremely time consuming. We therefore simulated the detection mechanism by which a trained researcher determines whether a specific voxel intensity belongs to the ROI or not. To this end, two experienced raters (MM, MCK) labeled 18 volumes with varying voxel-size and shape as described above. Since the purpose of this endeavor was to identify the detection mechanism of volume edges and since manual labeling of volumes with high resolutions is very time consuming, we choose to include only those volumes that have a resolution of 0.5mm isotropic or larger. For each volume, intensities were scaled to values between 0.0 and 1.0. Joint-volumes were calculated so that only those voxels that both raters agreed on were included in the volume. See Table 2 for the volumes and the agreement (Dice coefficient scores; DCS) between the two raters, which served as input to generate the decision-boundary used for our analyses as described in the section *Liberal and joint rater* below.

**Table 2:** Mask volumes for rater 1 and 2 (R1 and R2) and their conjunct mask. Labeled volumes were oriented diagonal to the slice axes and were reconstructed from one simulated ellipsoid at different resolutions (x, y, z). Voxel size indicates the fraction of the ground truth volume (164.9mm^3^) represented by 1 voxel. DCS show a strong overlap (>0.87) between the two raters.

Resolution			Voxel size	R1	R2	conjunct	
X	Y	Z	%Volume	Volumes		Volume	DCS
0.5	0.5	0.5	0.08	189.8	183.1	182.8	0.98
0.5	0.5	0.6	0.09	192.0	182.6	182.4	0.97
0.5	0.5	0.75	0.11	190.1	182.6	182.6	0.98
0.5	0.5	0.9	0.14	196.2	185.0	185.0	0.97
0.5	0.5	1.0	0.15	194.3	185.5	185.5	0.98
0.5	0.5	1.5	0.23	210.0	194.6	194.3	0.96
1.0	1.0	1.0	0.61	222.0	200.0	200.0	0.95
1.0	1.0	1.2	0.73	217.2	193.2	193.2	0.94
1.0	1.0	1.5	0.91	238.5	213.0	213.0	0.94
1.0	1.0	1.8	1.09	235.8	216.0	216.0	0.96
1.0	1.0	2.0	1.21	242.0	216.0	216.0	0.94
1.0	1.0	3.0	1.82	249.0	222.0	222.0	0.94
2.0	2.0	2.0	4.85	288.0	256.0	256.0	0.94
2.0	2.0	2.4	5.82	316.8	259.2	259.2	0.90
2.0	2.0	3.0	7.28	336.0	276.0	276.0	0.90
2.0	2.0	3.60	8.73	374.4	345.6	345.6	0.96
2.0	2.0	4.00	9.70	416.0	320.0	320.0	0.87
2.0	2.0	6.00	14.55	216.0	264.0	216.0	0.90

#### Liberal and joint rater

Next, we analyzed each rater's individual mask that included the intensities that were labeled as part of the volume. First, for each resolution, a joint mask was created consisting of voxels that were labeled by both raters (joint rater). Next, we fit a logistic function to the binomial labeling data of each mask using maximum likelihood maximization. The probability that voxel intensity (*i*) was labeled as part of the volume was calculated in the following way:

$$P_{volume}=\frac{1}{1+e^{-(\beta 0+\beta 1.i)}}$$

For each rater's mask and each joint mask, regression coefficients (β*0*, β*1*) were averaged across the different resolutions and used as psychometric curves. These psychometric curves represent the mean sensitivity and discriminability for the individual raters and the joint rater. For simulation purposes, we used the psychometric curves of the most liberal rater and the more conservative joint rater. These psychometric curves were used to determine the probability that, giving the intensity level, an edge-voxel would be included in the volume.

#### Optimal rater

**Fig 3:**
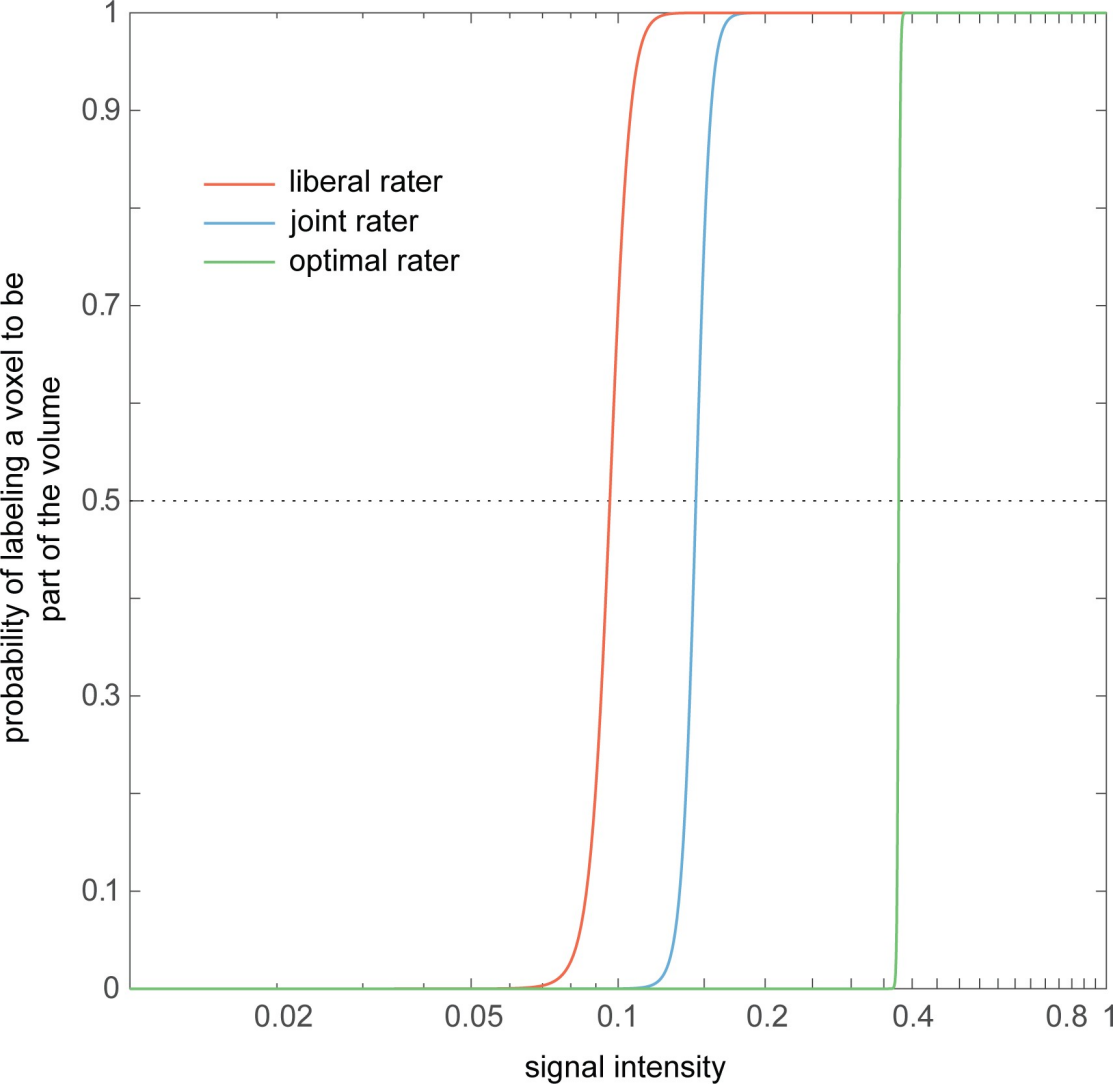
Psychometric curves used to simulate detection-thresholds and sensitivity in labeling procedures. Psychometric curves represent the probability (y-axis) that a voxel will be labeled as part of the ellipsoid given its signal intensity (x-axis). Colors represent the liberal human rater (red), the joint-labeling of two human raters (blue), and as a reference, the (average) optimal rater (green). Values are presented on a logarithmic scale.

In addition to the probabilistic raters described above (*liberal* and *joint* raters), we created an optimal rater. For the optimal rater, the intensity value cutoff for each volume was set to a level by which the number of included voxels resulted in a volume that approximated the predefined volume of the ellipsoid closest. To this end, we calculated the proportion of volume that a ground truth ellipsoid covered of a cube with the size of the FoV. Next, for each reconstructed image, we calculated the empirical cumulative density function and interpolated the intensity level where the proportion of the reconstructed volume resulted in the closest approximation of the expected proportional volume to the ground truth. In Fig 3 we illustrate the psychometric curve of the optimal rater, similar to the procedure described for the probability raters.

### Volume and shape similarity

For each rater (liberal, joint, optimal), and each reconstructed ellipsoid, we calculated the volume by calculating the number of voxels times the voxel size. In addition, the percentage deviation in volume, compared to the ground truth volume of the ellipsoid was calculated. Volume estimates do not provide information on potential differences in shape. For example, it is feasible that a mask-volume at a low resolution is close to the ground truth volume of the ellipsoid, although the shape of the mask is resembling a cube more than an ellipsoid (see also Fig 2). Therefore, for each reconstructed ellipsoid, we up-sampled the volume to the highest resolution (0.1 x 0.1 x 0.1mm) and compared the labeled mask with that of an optimal rater reference mask at that highest resolution (0.1 x 0.1 x 0.1mm). Up-sampling was done using an affine spatial transformation that was applied to each volume using a resampler structure in MATLAB (version2016b; MathWorks, Natick, MA) with a nearest neighbor interpolation kernel to preserve as much as possible the coarser resolution information. Volumes of the final resampled ellipsoids did not differ from the original low-resolution volumes, confirming that there were no additional resampling effects. DCS were calculated by two times the overlap (joint) between the reference volume and the up-sampled volume, divided by the sum of the two volumes [30]. For ellipsoids that were generated with slice thickness factors of 1.2 and 2.0, matrix sizes were uneven (respectively, 15 and 9), leading to mismatch between the reference and up-sampled matrix. To control for mismatch, the matrix size of the reference volume was adjusted to match the size of the up-sampled matrix without changing the ellipsoid volume.

## Results

We simulated the MR reconstruction of 100 mathematical ellipsoids at 30 different resolutions (five in-plane resolutions, and six slice thicknesses) and three different orientations relative to the slice-axis. For each reconstructed ellipsoid, the edge voxels were labeled as part of the volume or not, using psychometric curves of a liberal and joint rater, simulating the detection process of a manual rater. As a reference, we simulated an optimal rater with a labeling threshold which resulted in volumes that were as close as possible to the ground truth volume.

### Volumes

**Fig 4:**
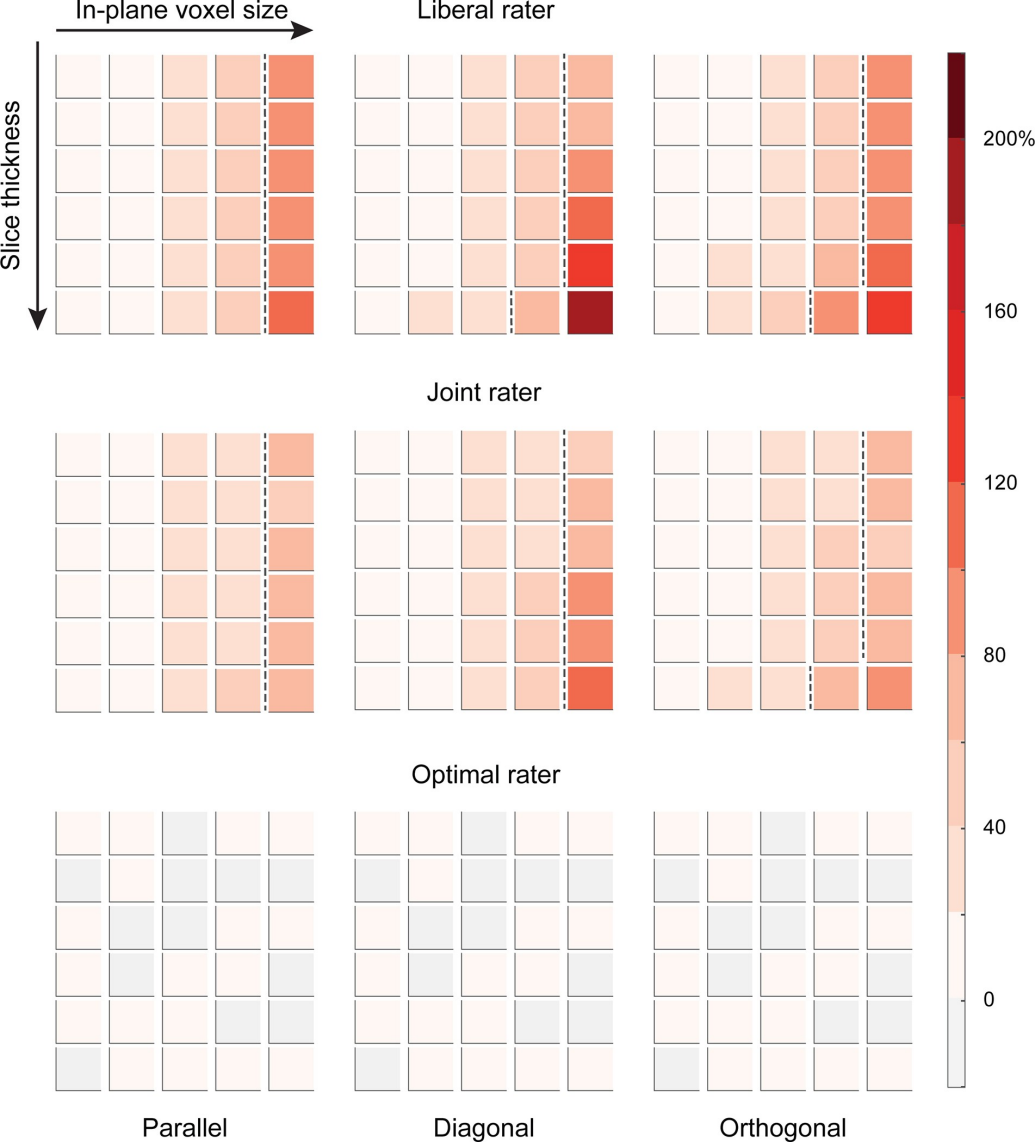
Overview of the average percentage deviation in volume for the simulated ellipsoids (n = 100), labeled according to a liberal, a joint, and an optimal rater. Columns refer to the orientation of the ellipsoid (parallel, diagonal, and orthogonal to the slice axes). Each block of squares represents sizes and shapes as depicted in Fig 2, with increasing in-plane voxel size (0.1 to 2.0mm) from left to the right, and increasing anisotropy (1x to 3x the in-plane voxel size) from top to bottom, with each top row being isotropic. Color intensity indicates the average percentage deviation in volume. Blocks to the right of the dotted lines represent deviations in volume that are > 57% of the ground truth, corresponding in DSC scores < 0.75, as shown in Fig 5 below (see S1 Fig for the relationship between volume deviations and DCS).

For each orientation and each rater, we present the mean percentage deviation in volume compared to the ground truth volume (see Fig 4). For the probability raters, the 95% confidence intervals (CI) fell between 0.007 and 8.13, whereas the CIs for the optimal rater were very small and fell between 0 and 1.776 x 10^−15^. For both the simulated liberal and joint raters, proportional deviations from the ground truth were small (less than 7%) for the high-resolutions (in-plane 0.1 x 0.1mm), independent of the anisotropy factor. For ellipsoids with an orientation parallel to the slice axis, deviations for in-plane resolutions of 0.2 x 0.2mm were still considered small, with deviations from the ground truth that were below 10%.

For voxels sizes larger than 0.2 x 0.2mm in-plane, deviations in volume increased markedly for the liberal and joint raters. For instance, a volumetric overestimation of 11% for the ellipsoid used in this study was observed for the 0.2 x 0.2 x 0.6mm in orthogonal orientation. At an in-plane voxel size of 2.0 x 2.0mm, deviations were larger for ellipsoids having a diagonal orientation, with a proportional increase of 206% when using a resolution of 2.0 x 2.0 x 6.0mm. As a result of the study design, for the optimal rater, volumes were close to the ground truth, for all voxel sizes.

### Shape similarity

For all raters, DCS were > 0.90 for all shapes and sizes for in-plane voxel resolutions of 0.1 x 0.1 and 0.2 x 0.2mm. They became lower for resolutions exceeding 0.5 x 0.5mm in-plane, with DCS smaller than 0.7 for the liberal rater for volumes with an in-plane resolution of 2.0 x 2.0mm.

Anisotropy negatively affects DCS. This was particularly the case for coarser resolutions, where the DCS scores for the liberal rater fell below 0.7 for ellipsoids with an orientation orthogonal to the slice axes, at resolutions of 1.0 x 1.0 x 3.0mm and lower.

The joint rater showed DCS <0.7 for an anisotropy factor of 1.8 for volumes with an in-plane resolution of 2.0 x 2.0mm, for all orientations. For the orthogonal orientation, the effect was more pronounced with DCS <0.7 at an anisotropy factor of 1.2 and an in-plane resolution of 2.0 x 2.0mm.

For all orientations, the optimal rater showed DCS < 0.7 for the most anisotropic voxel sizes for the in-plane 2.0 x 2.0mm resolution (factor 3), while DCS worsened for the diagonal orientation, where they were < 0.7 for an anisotropy factor of 1.8 for the 2.0 x 2.0mm resolutions.

### Same size, different shape

**Fig 5:**
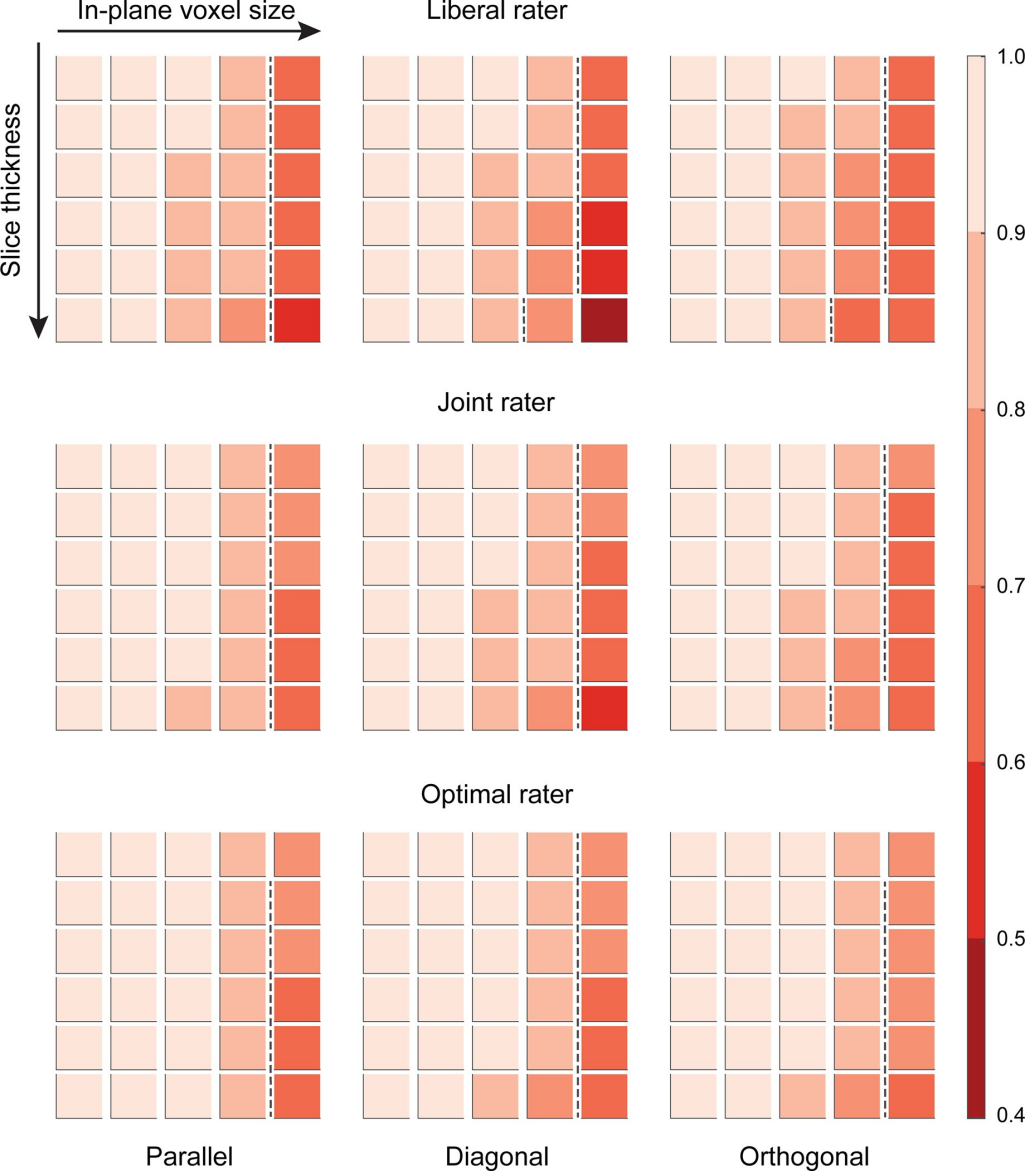
Overview of average shape similarity between the simulated ellipsoids and a high-resolution reference ellipsoid of 0.1 x 0.1 x 0.1mm. Volumes (n = 100) by all three raters were up-sampled to the reference resolution to create a conjunct mask between the reference volume and the labeled volume. Columns refer to the orientation of the ellipsoid (parallel, diagonal, and orthogonal to the slice axis). Each block of squares represents sizes and shapes as depicted in Fig 2, with increasing in-plane resolution (0.1 to 2.0mm) from left to the right, and increasing anisotropy (1x to 3x the in-plane voxel size) from top to bottom, with each top row being isotropic. Color intensity indicates the average shape similarity expressed in DCS. Blocks to the right of the dotted line represent DCS scores < 0.75.

Figs 4 and 5 show that raters, independent of their thresholds (*liberal*, *joint*) increasingly overestimate the volume of a ~165.0mm^3^ ellipsoid when resolution decreases (larger than 0.5mm isotropic) and anisotropy increases. In addition, for coarser resolutions, the shape of the volumes further deviates from an ellipsoid, with increasing size and anisotropy of the voxels (see Fig 2).

Note, however, that for the above used resolutions examined, anisotropic voxels have larger volumes compared to isotropic voxels. To compare voxels that only differ in shape, we included an additional factor of anisotropy of 8 times the in-plane resolutions for the 0.1 x 0.1mm and 0.5 x 0.5mm in-plane resolutions, and compared those to the volumes rated at isotropic voxels with the same size (resp. 0.2 x 0.2mm and 1.0 x 1.0mm). For clarity, comparisons are only done for the labeled volumes by the joint rater, which aligns with our previously published studies (e.g., [31–37].

**Fig 6:**
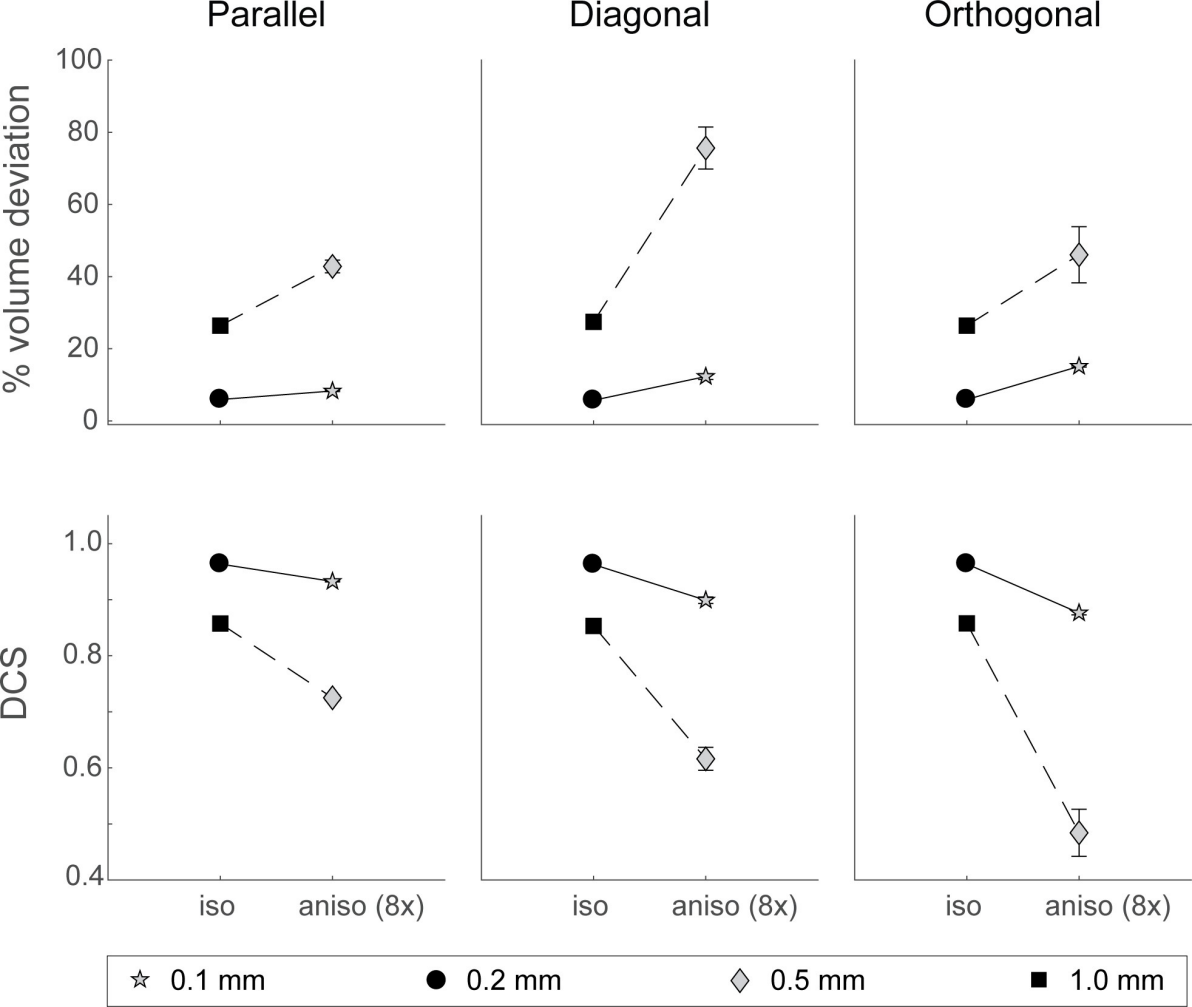
Effects of anisotropy. Comparison between volume and DCS for isotropic (0.2 x 0.2 x 0.2mm and 1.0 x 1.0 x 1.0mm) and anisotropic (0.1 x 0.1 x 0.8mm and 0.5 x 0.5 x 4.0mm) voxels of the same volume, labeled by the joint rater. Horizontal panels reflect orientations. Error bars indicate standard deviations from the mean.

As can be seen in Fig 6, the effect of anisotropy on the deviation in volume and shape similarity is moderate when resolutions are relatively high (0.1mm anisotropic vs. 0.2mm isotropic). However, for the high in-plane voxel size of 0.5 x 0.5mm, the 8-fold anisotropy causes severe overestimations of volumes and an greater mismatch of the ellipsoid shape, compared to the isotropic coarser resolutions of 1.0 x 1.0 x 1.0mm. Interestingly, the deviations in volume is largest for ellipsoids with an orientation diagonal to the slice axis, while shape deviates more for ellipsoids with an orientation orthogonal to the slice axis.

### The selective influence of voxel-size and shape on similarity

**Fig 7:**
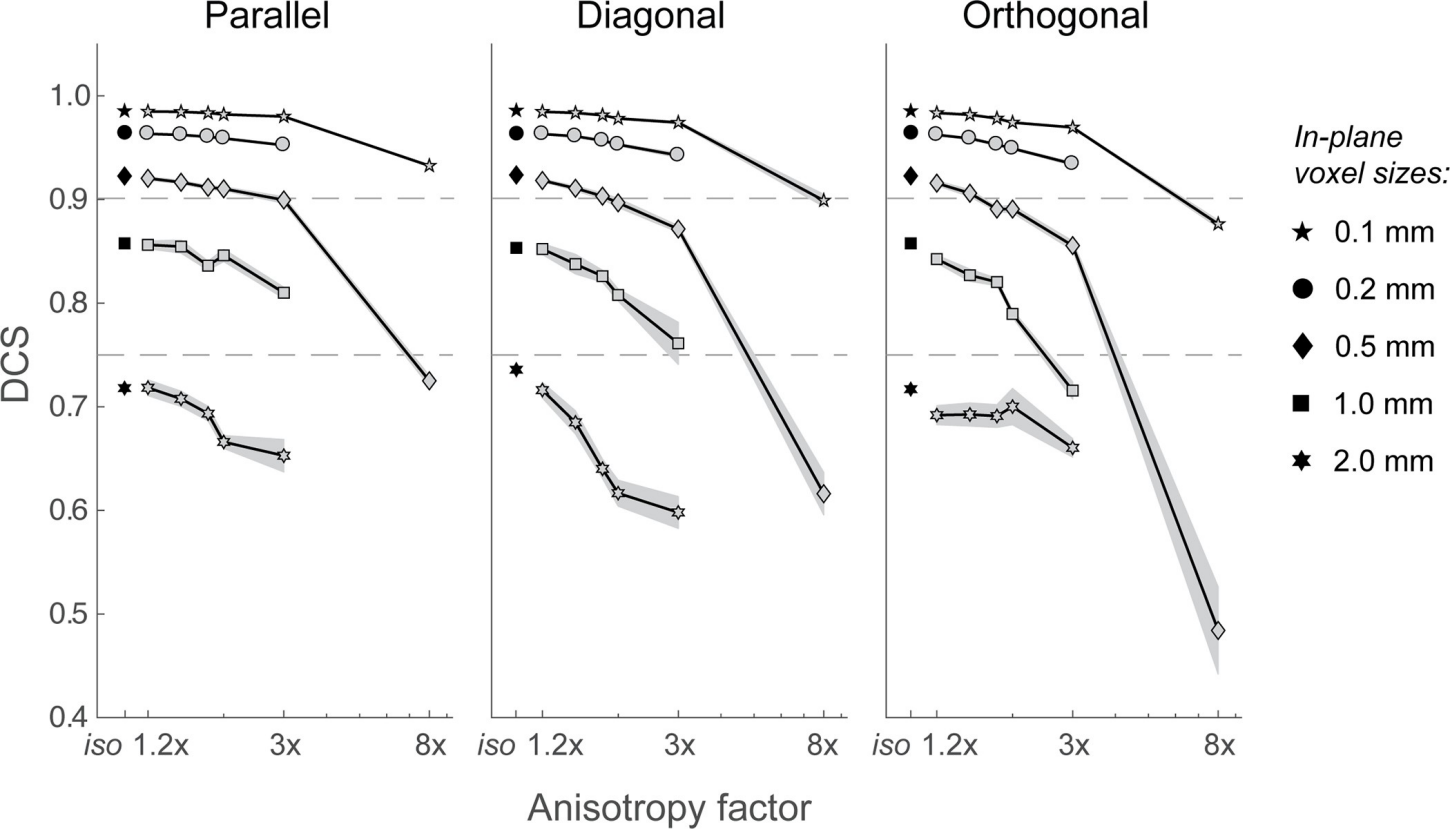
Similarity between volumes rated by the joint rater and the high-res (0.1mm) reference volume rated by the optimal rater. A DCS of 0.75 is considered the lower end of acceptable values [38]. DCS > 0.90 are considered ideal. Different marker symbols refer to the mean DCS for the different in-plane voxel sizes (0.1, 0.2, 0.5, 1.0 and 2.0mm). Isotropic voxel-size have a (rounded) relative volume of respectively 0.0006%, 0.005%, 0.08%, 0.6%, 5% of the ground truth ellipsoid volume (~165.0mm^3^). The grey area represents standard deviations from the mean. Note that the standard deviations increases for the most anisotropic resolutions due to the increasing PVE for ellipsoids with large voxel sizes and increasing anisotropy. For visualisation purposes, anisotropy factors (x-axis) are plotted on a logarithmic scale. Dashed lines indicate the 0.75 and 0.90 DCS values.

The effect of in-plane voxel-size and slice thickness on the volume and shape similarity of a structure is relative to the size of the ROI. To detect the borders of a ROI, the sample rate around the edges should be high enough to minimize the PVE, and allow accurate identification of the borders. Therefore, voxel sizes and slice thickness should be chosen so that volume and shape of the ROI will still approximate the ground truth. Since no guidelines are available on what is considered acceptable, we plotted the effects of voxel size and shape on shape similarity for the joint rater against the factor of anisotropy for each resolution (see Fig 7). We defined an arbitrary cut off at a DCS of 0.75 which is considered the lower end of acceptable values [38].

Fig 7 shows that using isotropic voxels smaller than 5% of the total volume of the ROI results in acceptably DCS > 0.75. However, when high accuracy is required (DCS > 0.90), the volumes of isotropic voxels should not exceed 0.08%, of the total volume. Anisotropy has relatively small effects when resolutions are < 0.6% of the total volume. However, when the voxel size accounts for >0.6% of the total volume, DCS become lower for large anisotropy factors (>3), particularly for orientations where the anisotropy is oriented orthogonal to the longer axes of the ROI (see orthogonal; 1.0mm, with an anisotropy of 3; Fig 7). Voxel sizes with a proportional size of 0.08% (0.5mm) of the total volume become unreliable with anisotropy factors of 8. For ellipsoid orthogonal to the imaging plane, the voxel volume should not exceed 0.005% of the total volume to compensate for anisotropy effects, in order to reach accuracy in the acceptable range (DCS > 0.75).

## Discussion

To quantify the effects of the size and shape of voxels on volume estimations and shape similarity of a small region of interest, we generated ellipsoids using different in-plane voxel sizes and varying slice thickness. These were subsequently labeled by simulated raters with different intensity cutoffs (liberal, joint, and optimal rater). For the *liberal* and *joint* raters, edge voxels were included based on a probability function derived from a liberal human rater and the joint (conjunct) labeling of two human raters, respectively. For the *optimal rater*, the decisions were simulated so that edge voxels were included until the labeled volume optimally approximated the ground truth.

As expected, both volume and similarity are affected by changes in the in-plane voxel size and slice thickness. For the ellipsoids used in this study, volume estimates consistently deviate more than 50% from the ground truth when slice thickness has a three-fold anisotropy for the commonly used resolution of 1.0 x 1.0mm in-plane voxel size. This effect was independent of the orientation of the ellipsoid (see Figs 4, 1.0 x 1.0 x 3.0mm). Isotropic voxels of 2.0 x 2.0 x 2.0mm or larger, resulted in volume estimates exceeding 170% of the ground truth. Similar results, although less pronounced, were observed for the joint rater.

High anisotropy factors have been reported in the literature and have been used to visualize a number of subcortical structures. For instance, to study the locus coeruleus, a small elongated structure in the brainstem, voxel sizes have been applied with anisotropy factors up to 7.7 (see Table 2 in [39]. For the substantia nigra, anisotropy factors up to of 8-times the in-plane resolution were used (see Table 2 in [6]. To investigate the subfields of the hippocampus, anisotropy factors up to 10.26 times the in-plane resolution were reported (see Table 1 in [40]. Additionally, clinical scans used to identify the STN or GPi for surgical planning report anisotropy factors up to 5 times the in-plane resolution (e.g., see Table 1 in [41] and Table 1 in c[42] and 4 times the in-plane resolution (see Table 1 in [10], respectively.

Results for the ellipsoid used in this study show that the size and shape of voxels affect the labeling results, particularly for coarser resolutions with voxel sizes over 5% of the ROI volume. Anisotropy factors of 1.2 to 2 times the in-plane resolutions have relatively small effects on volume estimates and DCS for in-plane resolutions that are relatively high and in which voxel volume is smaller than 0.6% of the ROI volume. However, for voxels with a volume exceeding 0.6% of the ROI volume, anisotropy can result in substantial deviations from the ground truth, especially when the in-plane resolution decreases. These effects interact with the orientation, most pronounced, when the anisotropy is oriented orthogonal to the longest axis of an ellipsoid volume.

### Effects of orientation

Interestingly, when anisotropy increases, the effect of orientation was most pronounced in ellipsoids with an orientation diagonal to the slice axis, showing increasing deviations in both volume estimates and shape similarity for in-plane resolutions of 5% of the ROI volume (2.0mm; see Figs 4, 5 and 7). For extreme anisotropy factors (8x in-plane resolution), effects of orientation on shape similarity was largest in ellipsoids with an orientation orthogonal to the slice axes (see Fig 6). These effects suggest that the orientation of structures of interest should be taken into account when defining the voxel size and the level of anisotropy. For instance, for a structure like the STN which has a diagonal orientation, choosing a voxel geometry with a large anisotropy factor might be quite detrimental.

### Effects of raters

As illustrated in Fig 5, the effects are larger when a liberal segmentation strategy is applied (liberal vs. joint rater). This finding attests the importance to use conjunct or joint ratings of multiple raters in manual labeling studies. However, even for a joint rater, relatively low resolutions (>5%, here: in-plane of 2.0mm) affect the volume estimates and DCS as such, that results might become unreliable, especially when anisotropy increases (see Fig 5).

Interestingly, all deviations shown are driven by an overestimation of the ellipsoid volume (see Figs 4 and 6). This is already reflected in the manual labeling process by the two raters R1 and R2 (see Table 1) that shows overestimations of the ellipsoid volume. This overestimation is even apparent in the joint volume estimations, which is by definition more conservative than a single rater. Possibly, these overestimations might reflect a general human perceptual bias to include ambiguous intensity levels into a volume of interest.

### Implications for understanding structure and function of the human subcortex

Voxel geometry matters for the volumetric and shape estimates of small regions of interest such as small nuclei in the human subcortex. Here, we chose to simulate small volumes which are representative for many small nuclei deep in the brain including the STN and GPi. Our present findings indicate, that the chosen voxel geometry clearly impacts the labeling accuracy of small subcortical structures. The effects of voxel shape and voxel size have to be taken into account when acquiring MRI data. Given the clinical application of anisotropic scanning strategies for surgical planning, our findings are of importance for the clinical neurosciences and neurosurgery.

### Limitations

We simulated the labeling procedure using psychometric curves of human raters (Fig 3) while taking into account the probability that a voxel will be labeled as part of the volume given its intensity. However, it is important to acknowledge that our results are based on simulated noise-free MR images, in which the SNR exclusively depends on the chosen resolution and simulated variation in motion. Factors such as physiological properties of the tissue, temperature, air or blood which are known to negatively affect SNR flow [5] and further complicate the visualization of small brain structures, were not simulated. Furthermore, tissue can differ in susceptibility to the different properties of the MR signal (e.g., T_1_ vs T_2_* signal decay, see for example [43,44] that result in different contrasts depending on the used scan sequence. As such, contrast-to-noise is typically important as well, when labeling small regions in the brain. Although we acknowledge the difference between real MR images and our simulated data, in the current study we opted to focus on the effect of PVE on the labelling accuracy due to choices in voxel-size and shape.

In addition to the rather ideal MR signal, the shape of the region of interest was close to ideal as well. Given the fact that anatomical structures are often more variable and irregular in shape, it is hard to predict how results would relate to effects of voxel size and slice thickness on volume and shape estimations of less ideal structures. Nevertheless, the quantification of the deviations provides a ballpark estimation of the limits when using (anisotropic) voxel sizes.

It is clear that voxel size and slice thickness can influence the visualization process of anatomical target structures. Extrapolation of the results to specific target shapes remains challenging also in view of the additional dependence on the specific anatomy and MRI characteristics of individual target structures as well as the characteristics of neighboring structures, and available MRI contrasts.

With respect to the labeling procedure itself, one could argue that human raters will, at times, deviate from these psychometric curves due to factors not captured in the simulation itself and which may interact with voxel size and shape. Another limitation is that we did not include any prior shape information of the ellipsoid. However, our study was designed to assess the effects of voxel size and shape, and therefore the potential effects of shape priors lies beyond the scope of this simulation study. To determine potential effects of the variability between individual raters, we presented the results for both raters as well as the results for an optimal rater, which already revealed clear effects of voxel size and shape on the labeling procedure.

## Conclusions

*Size* and *shape* of a voxel measured with magnetic resonance imaging (MRI) affect the ability to visualize small brain nuclei. In this study, we demonstrate the selective influence of voxel geometry by reconstructing simulated ellipsoid structures with voxels varying in shape and size. As expected, the results show that larger voxels (i.e., coarser resolution) and increasing anisotropy result in increased deviations of both volume and shape measures of the simulated structures of interest. To ensure deviations occur within the acceptable range (DCS > 0.75, corresponding to < 57% volume deviation), the volume of isotropic voxels should not exceed 5% of the total volume of the region of interest. When high accuracy is required (DCS > 0.90, corresponding to a < 19% volume deviation), the volumes of isotropic voxels should not exceed 0.08%, of the total volume. Finally, when large anisotropic factors (>3) are used, under the worst case of the ellipsoid being orthogonal to the slice axes (i.e. having its long axis in the imaging plane), the voxel volume should not exceed 0.005% of the total volume to compensate for anisotropy effects, in order to reach accuracy in the acceptable range (DCS > 0.75, corresponding to >57% volume deviation; see S1 Fig for the relationship between volume deviations and DCS.

## Supporting information

S1 Fig Relationship between volume deviations and DCS. Volume deviations and the accompanying DCS scores are plotted for all simulated ellipsoids that were labeled according to a liberal (red) and a joint rater (blue). The solid line represents the exponential function that was fitted to the data and was used to find the volume deviations corresponding to the ideal (DCS > 0.90) and acceptable (DCS > 0.75) range.
(TIF)
