# Supplementary figures and images for: Size and shape matter: The impact of voxel geometry on the identification of small nuclei
Source: PLoS One. 2019 Apr 12;14(4):e0215382. doi: 10.1371/journal.pone.0215382 (PMC6461289; doi:10.1371/journal.pone.0215382)

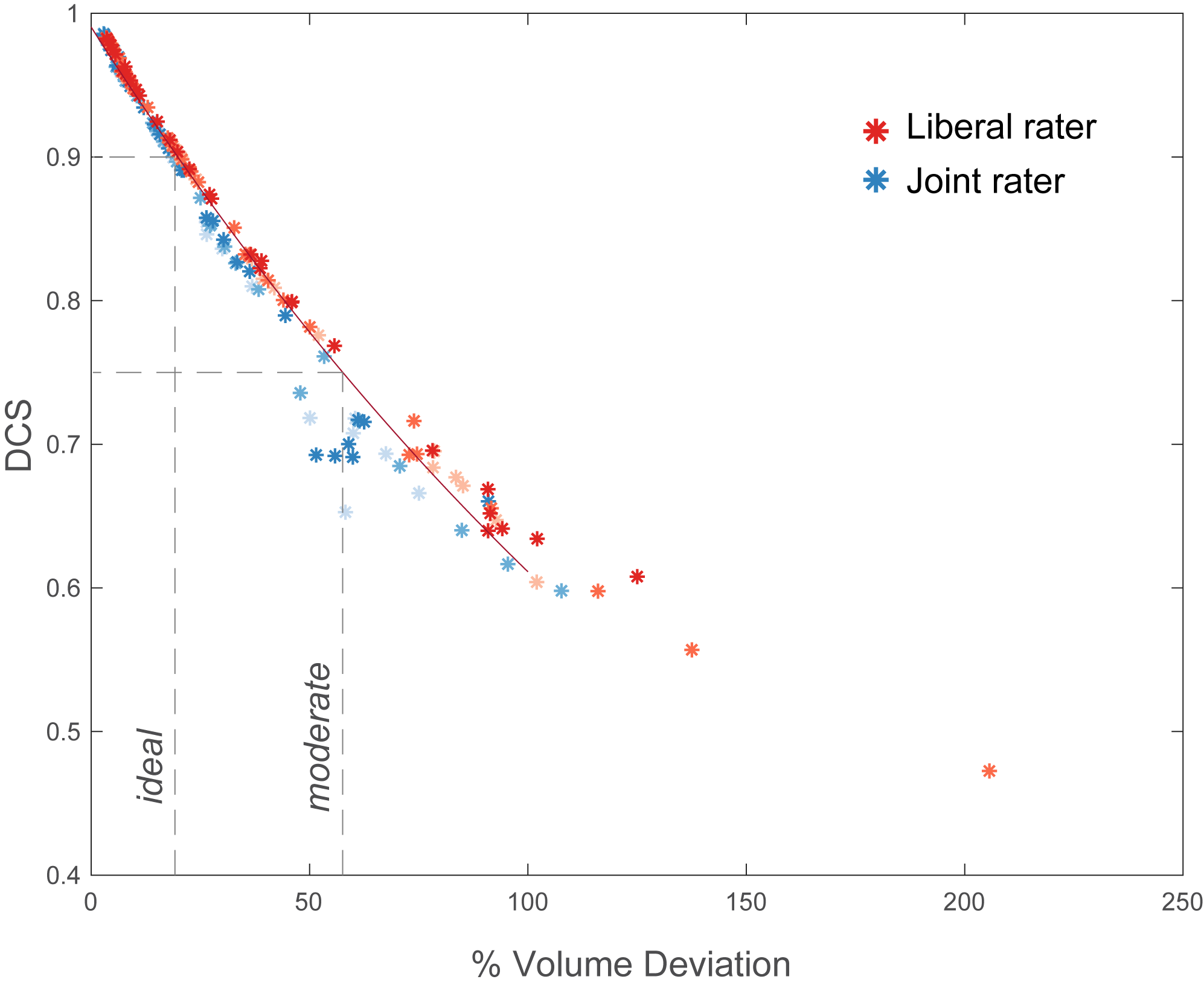

Supplement: S1 Fig — Volume deviations and the accompanying DCS scores are plotted for all simulated ellipsoids that were labeled according to a liberal (red) and a joint rater (blue). The solid line represents the exponential function that was fitted to the data and was used to find the volume deviations corresponding to the ideal (DCS > 0.90) and acceptable (DCS > 0.75) range. (TIF) [file pone.0215382.s001.tif]
